# Impact of Superparamagnetic Iron Oxide Nanoparticles on Vocal Fold Fibroblasts: Cell Behavior and Cellular Iron Kinetics

**DOI:** 10.1186/s11671-017-2045-5

**Published:** 2017-04-20

**Authors:** Marina Pöttler, Anna Fliedner, Eveline Schreiber, Christina Janko, Ralf Philipp Friedrich, Christopher Bohr, Michael Döllinger, Christoph Alexiou, Stephan Dürr

**Affiliations:** 1 Department of Otorhinolaryngology, Head and Neck Surgery, Section of Experimental Oncology and Nanomedicine, Else Kröner Fresenius Stiftung-Professorship, University Hospital Erlangen, Erlangen, Germany; 2 Department of Otorhinolaryngology, Head and Neck Surgery, Division of Phoniatrics and Pediatric Audiology, University Hospital Erlangen, Erlangen, Germany; 3 Department of Otorhinolaryngology, Head and Neck Surgery, Section of Experimental Oncology and Nanomedicine (SEON), University Hospital Erlangen, Glückstraße 10a, 91054 Erlangen, Germany

**Keywords:** Vocal fold, Magnetic tissue engineering, Superparamagnetic iron oxide nanoparticles

## Abstract

**Purpose:**

The voice is the most important instrument of communication. Tissue defects in the vocal fold (VF) area lead to serious reduction in quality of life, but thus far, no satisfactory VF implant exists. Therefore, we aim to establish a functional VF implant in a rabbit model by magnetic tissue engineering (MTE) using superparamagnetic iron oxide nanoparticles (SPION). Hence, iron quantification over time as well as cell behavior studies upon SPION treatment are of great importance.

**Methods:**

Rabbit VF fibroblasts (VFF) were treated with different concentrations of SPIONs (20, 40, and 80 μg/cm^2^), and iron content was examined for up to 40 days using microwave plasma-atom emission spectroscopy. The effects of SPION treatment on VFF (adhesion, spreading, and migration), which are important for the formation of 3D structures, were tested.

**Results:**

Cellular SPION quantification revealed that there was no residual iron remaining in VFFs after 40 days. SPIONs had a dose-dependent effect on cell adhesion, with good tolerability observed up to 20 μg/cm^2^. Migration and spreading were not significantly influenced by SPION treatment up to 80 μg/cm^2^.

**Discussion and Conclusion:**

To develop 3D structures, cell behavior should not be affected by SPION uptake. After 40 days, cells were free of iron as a result of metabolism or rarefication during cell division. Cell functions including adhesion, spreading, and migration were proven to be intact in a dose-dependent manner after SPION treatment, suggesting a safe usage of MTE for voice rehabilitation. Our results thus constitute a solid basis for a successful transfer of this technique into 3D constructs, in order to provide an individual and personalized human VF implant in the future.

## Background

The voice is the main instrument of communication; therefore, losing one’s ability to speak is highly associated with significant loss in quality of life [1]. Tissue defects of the vocal folds occurring after trauma or diseases such as cancer predominantly cause potent problems for patients, since they are additionally confronted with a communication problem together with their serious illness. Thus far, neither satisfying vocal fold transplants nor implants exist, and the adequate test systems for the biology and biomechanics of the vocal fold (VF) are lacking [2]. Implants or injections of regenerative biomaterial are succinctly capable to recover the VF, but most of them are either rejected or quickly metabolized [3, 4]. Consequently, tissue engineering of the VF is an urgent clinical need. An engineered VF tissue, which is able to restore voice and complementary preserve a shutoff against the airway lumen, would support overcoming this deficiency.

Basic research approaches have already been proposed in the area of tissue engineering of the VF [5], including three-dimensional adipose-derived stem cell implantation [6] or using scaffolds colonized with suitable cells [7, 8]. But so far, these implants cannot be directly compared to VF tissue, and not enough improvement has been made on the subject of re-establishment of structure and physiological function [9]. Considerable efforts have also been made in the field of tissue engineering when using scaffold material to generate 3D cell construct or tissue structures, including matrigels or polymeric constructs [10, 11]. But these scaffolds also have their restrictions and are rather unpractical, since they may act as a transport barrier for nutrients and allow metabolic waste increase, inhibiting cell proliferation and cell-cell interactions, which are very important for tissue formation. This consequently leads to a poorer outcome of a 3D construct [12].

Magnetic nanoparticles are used in a broad spectrum of medical applications [13]. Particularly, they have been utilized for drug delivery [14–19] or used for magnetic resonance imaging as contrast media [20]. Moreover, magnetic nanoparticles have been established for cell sorting and microtissue arrangement by applying magnetic fields [21]. Using magnetic force for tissue engineering, so-called magnetic tissue engineering (MTE) has been tested in several applications, such as assembly of skeletal muscles and of small diameter blood vessels, bone tissue formation, or stem cell differentiation [22–26]. The basic concept includes loading of cells with magnetic nanoparticles. By doing so, cells become magnetizable and can then be controlled and guided under magnetic force, which leads to formation of 3D-cell constructs within hours.

Since there is limited access to disease-free human VFs, we used a rabbit-VF tissue. This approach is feasible, because the structure of VF and predominantly the *lamina propria* of humans and rabbits resemble each other closely [27]. In this study, we investigated whether SPIONs, which are used for magnetic tissue engineering, interfere with cell behavior including adhesion, spreading, or migration, which are fundamental for 3D tissue formation. Furthermore, we analyzed iron content in VF fibroblasts (VFF) for up to 40 days. This is particularly important because nanoparticles should not act as a vibration obstruction within a bioengineered vocal fold.

## Methods

### Materials

Ringer’s solution was purchased from Freseniun-Kabi, Bad Homburg, Germany; amphotericin B was from Biochrom, Berlin, Germany; Dulbecco’s modified Eagle’s medium (DMEM), phosphate-buffered saline (PBS), L-glutamine, fetal calf serum (FCS), trypsin, and penicillin/streptomycin were from Life Technologies, Darmstadt, Germany; paraformaldehyde (PFA) (CH_2_O), ethanol (C_2_H_5_OH), sodium hydrogen phosphate (Na_2_HPO), potassium hexacyanoferrate (K_4_[Fe(CN)_6_]), hydrochloric acid (HCl), sodium citrate (trisodium citrate-dihydrate C_6_H_5_Na_3_O_7_2H_2_O), and crystal violet were purchased from Carl Roth, Karlsruhe, Germany; mouse anti rat CD90:FITC antibody was from BioRad, Hercules, CA, USA; Triton X-100, collagenase, DNAse non-essential amino acid solution (NEAA), and lauric acid were from Sigma-Aldrich, Taufkirchen, Germany. In all experiments, double-distilled water was used.

### Superparamagnetic Iron Oxide Nanoparticles (SPIONs)

SPIONs were synthesized and extensively characterized at the Section of Experimental Oncology and Nanomedicine, SEON, as previously described in [14, 28]. SPIONs were synthesized by co-precipitation in aqueous media. Briefly, Fe(II) chloride and Fe(III) chloride salts were dissolved at a molar ratio of 2:3 in H_2_O ddist. Under stirring, 25% NH3 solution was added to precipitate Fe2O3/Fe3O4 particles. After washing the centrifuged particles with 1.3% NH3 multiple times, an aliquot of the SPION suspension was mixed with lauric acid and heated to 90 °C under stirring. After cooling, the particles were covered with a coating of bovine serum albumin. Lauric acid-coated SPIONs were incubated 1:1 (*v*/*v*) with a 10% (*w*/*v*) bovine serum albumin (BSA) solution and filtered through a 0.22-μm CME sterile syringe filter (Carl Roth, Karlsruhe, Germany). For physicochemical characterization, we completed several methods including transmission electron microscopy (Philips CM30 TWIN/STEM; Philips Analytical, Eindhoven, the Netherlands), dynamic light scattering (hydrodynamic diameter 132.4 nm), and zeta potential analysis (−25.37 mV) (Zetasizer ZLS 380; Nicomp, Port Richey, FL, USA) or diffuse reflectance infrared spectroscopy (Excalibur FTS 3100 Spectrometer; Varian, Walnut Creek, CA, USA) [14].

### Primary Cell Culture

Isolation and cultivation of rabbit VFF were performed according to Suehiro and colleagues [29]. Briefly, larynges of New Zealand White rabbits (Charles River, Sulzfeld, Germany) were extracted post-mortem*.* First, the lamina propria was removed from the VF and minced into little fragments. Then, pieces were digested via DNase and collagenase at 37 °C for 1 h. To obtain VFFs, cells were strained through a 40-μm filter and transferred in a well of a 6-well plate with 2 mL DMEM supplemented with L-glutamine (5 mM), 10% FCS, 1% amphotericin B, 1% penicillin/streptomycin, and 1× NEAA (full medium). For further cultivation, cells were kept at 37 °C in 5% CO_2_ humidified atmosphere. At 80 to 90% confluence, VFFs were detached using trypsinization and transferred first into T25 and later into T75 cell culture flasks (TPP, Trasadingen, Switzerland). Again, after reaching 80 to 90% confluence, cells were split every 2 to 3 days and 1 × 10^6^ cells were used for further cultivation and experimental onset in T75 culture flasks. Treatment of SPIONs in different concentration was tested for toxic effects on VFFs previously [30], revealing concentrations below 80 μg/cm^2^ as secure. To identify VFF, morphological standards were used such as spindle shape or cell size within 14 days. For further identification, CD90 expression was analyzed using flow cytometric measurements (Gallios flow cytometer, Beckman Coulter, Fullerton, CA, USA) according to the manufacturer’s instruction. Cells were harvested from rabbits sacrificed for the purpose of another study at the SEON, which obtained the ethical permission from the Animal Research Committee (Government of Mittelfranken; AZ 54.2532.1-21/11).

### Cell Adhesion

1.5 × 10^5^ cells were transferred into T25 culture flasks (TPP, Trasadingen, Switzerland), and after 24 h, different concentrations of SPIONs were added to cells (20, 40, and 80 μg/cm^2^). As controls, the equivalent amount of double-distilled water was used (SPION diluent). After another 24 h, cells were washed with PBS and detached with trypsin. Trypsinization was stopped by adding full media. Cells were counted via MUSE® cell analyzer, and 5 × 10^4^ cells per well were transferred in a 96-well plate. Cells were allowed to adhere for 5, 15, 30, or 45 min at 37 °C. Cells, which did not adhere, were washed away with PBS. After fixation with 4% PFA, cells were stained with 100-μl crystal violet (0.1%, filtered through a 0.2-μm filter) for 20 min at room temperature. After a three-time wash with PBS, crystal violet was extracted with 10% acetic acid and plates were measured with a multi-mode microplate reader (FilterMax F5, Molecular Devices, Sunnyvale, CA, USA) at 560 nm. [31]. Triplicate samples were used for each condition in at least three independent experiments.

### Cell Spreading

Cell spreading was analyzed using the same method as for adhesion except the extraction of crystal violet. Pictures were taken with a fluorescence microscope under bright field options (Axio Observer Z.1 with an ApoTome, Zeiss, Jena, Germany). At least nine pictures of each condition and each time point were taken, and by bordering every single cell, cell-area was analyzed via Image J software (NIH, Bethesda, MD, USA) [31]. For each condition, triplicate samples were used in at least three independent experiments.

### Cell Migration

After trypsinization, cells were counted using MUSE® cell analyzer and 3 × 10^4^ cells per well were seeded in 96-well plates and grown until 90% confluence. An equal scratch was then created in each well with a pipet tip. After “wounding”, cells were washed with PBS to remove cell debris and incubated with full medium. Using live-cell imaging (Incucyte®, Essen Bioscience, Michigan, USA), pictures of all wells were taken every 4 h for 48 h and analyzed for cell density using Incucyte® software (Essen Bioscience, Michigan, USA) [32]. Triplicate samples per each condition (control, 20, 40, and 80 μg/cm^2^) were used in at least three independent experiments.

### Cellular SPION Quantification

VFF (1 × 10^5^ cells) were transferred into 6-well plates and incubated with different concentrations of SPIONs (0, 20, 40, and 80 μg/cm^2^) for 24 h. Each condition was prepared in triplicates. Four days was the limit for an experimental set-up when using the selected cell number without having to split them. For this short-term investigation (up to 4 days), each condition was prepared separately and harvested on the day of interest. For long-term investigations (more than 4 days), this set was prepared once, and every 5 days, cells were split into two subpopulations. One half was cultured for further investigation; in the other half, cell number was measured using MUSE® system followed by iron content measurement by microwave plasma-atom emission spectroscopy (MP-AES). Briefly, the harvested cells were centrifuged (1000 rpm, 5 min), supernatant was discharged, and the pellet was kept at −20 °C until further processing. After thawing, 50 μl 65% HNO_3_ was added and suspension was heated at 90 °C for 10 min. 450 μl H_2_O ddist were added, and iron content was measured using MP-AES (Agilent 4200, Santa Clara, CA, USA). Iron content was normalized to the total number of cells within each cell pellet.

### Magnetic Cell Guidance in 2D

Cells pre-treated with different concentrations of SPIONs for 24 h were harvested and counted using MUSE® cell analyzer and transferred in a 6-well plate (at 1 × 10^6^ cells per well), which was placed on top of a magnet plate . This setup allowed to distinguish between magnet-exposed areas and magnet-free areas within one well. After 24 h, cells were washed three times with PBS and fixed with 4% PFA, followed by staining with either crystal violet or Prussian blue.

For crystal violet staining, cells were incubated with 500 μl crystal violet (0.1%, filtered through a 0.2-μm filter) for 20 min at room temperature followed by three times washing with PBS. For Prussian blue staining, potassium hexacyanoferrate (2%) was mixed with hydrochloric acid (2%) in 1:1 proportion and 500 μl were added to the wells for 30 min at room temperature. As a final step, cells were washed twice with PBS and finally with water. Pictures were taken with a microscope under bright field (Axio Observer Z.1, Zeiss, Jena, Germany). Quantitative analysis was performed using Image J software.

### Statistics

Data were analyzed using Student’s *t* test in Excel (Microsoft, Redmond, USA). All values are presented as mean ± standard deviation (SD) of at least three independent experiments. *p* values of *p ≤ 0.05 between control group and SPION-treated samples were considered statistically significant.

## Results and Discussion

One major advantage of MTE is that cells can be remote controlled by making them magnetic. This also enables us to manipulate the cell shape in 3D. But, considering the fate of cells in a 3D cell construct, in our case, a rabbit’s vocal fold cell behavior must not be affected by the bioengineering process. Especially using MTE, effects of nanoparticle treatment on cells must be clarified extensively. Consequently, we investigated the impact of SPIONs in VFF behavior, including cell adhesion cell spreading and cell migration.

### Adhesion, Spreading, and Migration

Cell adhesion is a prerequisite for VFF function regulation and cell communication. Notably, it is of central significance in the development and maintenance of tissues [33]. The mechanical cell-cell as well as cell-extracellular matrix (ECM) contacts can affect and regulate cell physiology [33]. In this study, we established that SPION concentrations up to 20 μg/cm^2^ did not significantly affect cell adhesion. Therefore, we assume that major cell regulatory processes are not affected at lower SPION concentrations. If cell behavior, e.g., migration or spreading, were altered by SPION uptake, this would consequently lead to suspect that major signaling pathways (e.g., PI3K) responsible for these actions were defective [34]. Therefore, we evaluated the spreading as well as the migratory behavior of VFF treated with SPIONs, showing that SPIONs had no significant impact on these VFF functions.

**Fig. 1:**
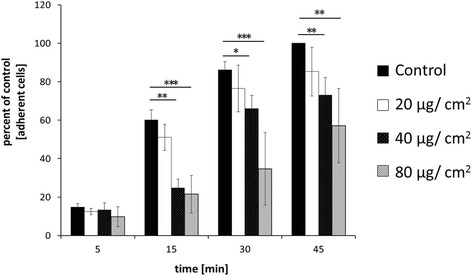
Cell adhesion in VFFs is altered by SPIONs in a dose-dependent manner. No significant changes were detected at first 5 min and when VFFs are treated with SPIONs up to 20 μg/cm^2^. Treatment with 40 and 80 μg/cm^2^ SPIONs resulted in significant decrease in VFF adhesion starting at 15 min. *p* values are indicated as *p ≤ 0.05, **p ≤ 0.001, ***p ≤ 0.0001

In order to measure cell adhesion, VFF were allowed to attach on the surface of cell-culture well-plates for 5, 15, 30, and 45 min. Adherent cells of the control group were used as reference (100%) after 45 min. No significant differences were observed between controls and cells treated with up to 80 μg/cm^2^ SPIONs after the first 5 min. However, compared to untreated controls, pre-treatment with 40 and 80 μg/cm^2^ of SPIONs significantly impaired attachment of SPION-loaded cells (Fig. 1). A concentration-dependent inhibition of VFF adhesion by SPIONs was observed.

**Fig. 2:**
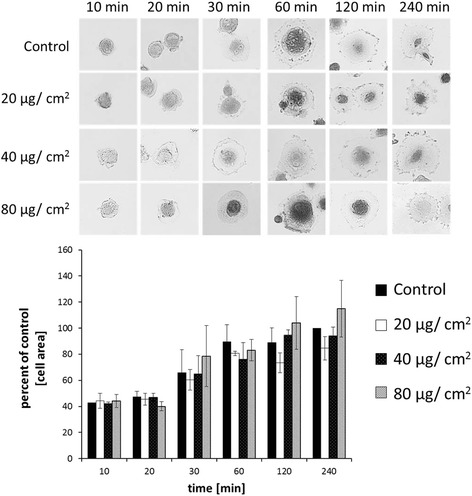
Spreading of VFF on cell culture plates was not significantly influenced by SPION treatment. Cell spreading was measured by evaluation of cell area (*upper section*). No significant differences were observed between values of control group and each SPION concentration for every time point, respectively (*lower section*).

To evaluate the possible influence of SPION uptake on VFF spreading, we analyzed the cell area of VFFs treated with 20, 40, and 80 μg/cm^2^ SPIONs for a specified period of time (10, 20, 30, 60, 120, and 240 min). No significant aberrations in cell spreading as compared to control were detectable at any tested SPION concentrations (Fig. 2).

**Fig. 3:**
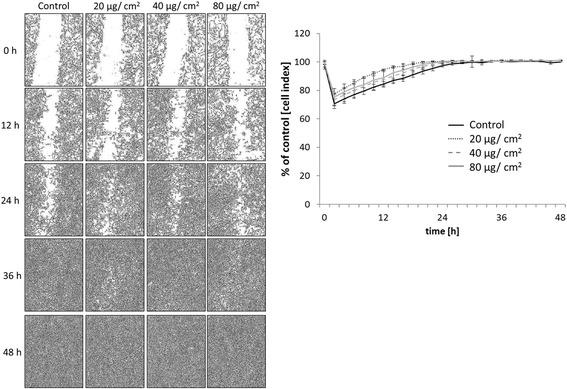
Cell migration analyzed using scratch assay. In the *left panel*, cell monolayer was wounded at 0 h and, after 48 h, this scratch was closed under each condition (control, 20, 40, and 80 μg/cm^2^ of SPIONs). In the *right panel*, confluence measured with Incucyte® revealed that cell migration was not significantly affected by treatment with different concentrations of SPIONs (as indicated). *p* > 0.05

The effect of different SPION concentrations (20, 40, and 80 μg/cm^2^) on VFF migration was evaluated using a scratch-wound assay. Results were compared to those obtained in samples treated with corresponding volumes of water as control. As shown in Fig. 3, SPIONs in concentrations up to 80 μg/cm^2^ did not decrease migratory behavior of VFF cells. All cells under different conditions were able to close the gap, independent of SPION concentrations. These data indicate that treatment of VFF cells with SPIONs up to 80 μg/cm^2^ does not affect cell migration.

### SPION Release Kinetics

**Fig. 4:**
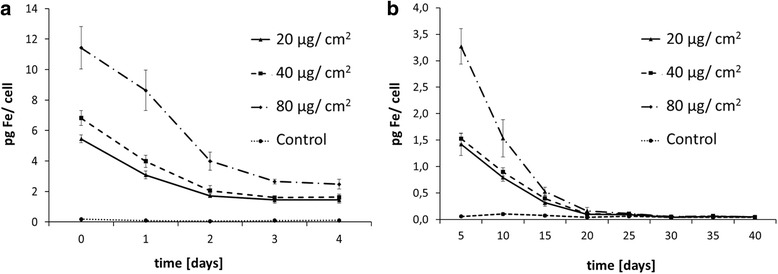
Iron clearance in long- and short-term measurements. Absolute iron content per cell was analyzed using MP-AES. **a** Iron content in VFFs was quantified daily for 4 days. Time point 0 represents the iron load 24 h after the addition of SPIONs. ***p ≤ 0.0001 (details in Table 1). (**b**) After 40 days, iron content in VFFs reached same level as the control group. *p ≤ 0.05, **p ≤ 0.001, ***p ≤ 0.0001 (details in Table 2)

As it is of particular importance to determine how long SPION remain in VFF, the iron content of SPION pre-loaded cells was analyzed using MP-AES at different time points. Two varying set-ups were selected, a short-term and a long-term experiment. In the first one, VFF were pre-treated with SPIONs for 24 h and, afterwards, the absolute iron content per cell was measured once a day. As expected, the highest iron content per cell was measured on the first day (i.e., after 24-h loading period), with a predominant decrease within the first 2 days (Table 1; Fig. 4a). After day four, cells were confluent and spitting was necessary to prevent apoptosis. Therefore, for continuous analysis until the point of no significant difference to the control was reached, a second long-term experiment was conducted, whereby the cells were split regularly. This experiment showed that the amount of iron in SPION pre-loaded samples returned to the control levels after day 40 (Table 2; Fig. 4b).

**Table 1:** Short-term cellular iron quantification

Mean pg Fe/cell	Day 0	Day 1	Day 2	Day 3	Day 4
20 μg/cm^2^	5.46 ± 0.25	3.09 ± 0.26	1.72 ± 0.13	1.45 ± 0.22	1.45 ± 0.21
40 μg/cm^2^	6.82 ± 0.49	3.98 ± 0.39	2.05 ± 0.34	1.60 ± 0.08	1.62 ± 0.19
80 μg/cm^2^	11.43 ± 1.38	8.63 ± 1.32	3.99 ± 0.59	2.65 ± 0.16	2.48 ± 0.16
Control	0.18 ± 0.05	0.09 ± 0.02	0.06 ± 0.02	0.09 ± 0.01	0.10 ± 0.01

*p* values of all data indicated are highly significant (*p* ≤ 0.0001) between values of control group and each tested concentration and among the respective SPION-treated samples between different days

**Table 2:** Long-term cellular iron quantification

Mean pg Fe/cell	Day 5	Day 10	Day 15	Day 20	Day 25	Day 30	Day 35	Day 40
20 μg/cm^2^	1.42 ± 0.21***	0.79 ± 0.07***	0.32 ± 0.07***	0.10 ± 0.01***	0.09 ± 0.01	0.05 ± 0.01	0.06 ± 0.01	0.05 ± 0.01*
40 μg/cm^2^	1.53 ± 0.09***	0.90 ± 0.08***	0.40 ± 0.07***	0.11 ± 0.01***	0.11 ± 0.02*	0.05 ± 0.02	0.07 ± 0.01**	0.05 ± 0.01
80 μg/cm^2^	3.27 ± 0.033***	1.54 ± 0.35***	0.53 ± 0.08***	0.16 ± 0.06***	0.11 ± 0.01**	0.06 ± 0.01	0.07 ± 0.01*	0.05 ± 0.01
Control	0.06 ± 0.02	0.10 ± 0.03	0.08 ± 0.03	0.04 ± 0.01	0.06 ± 0.03	0.05 ± 0.02	0.04 ± 0.01	0.04 ± 0.01

Data are significantly different between values of control group and each concentration for different days, when indicated as *p ≤ 0.05, **p ≤ 0.001, and ***p ≤ 0.0001

Regardless of all uptake studies performed in a variety of different cell types treated with SPIONs, it is so far unclear, which physicochemical properties of SPIONs are responsible for ideal uptake, in terms of MTE, in non-phagocytic cells and more importantly, how long do these particles remain within the cell. Especially when planning to implant the future bioengineered 3D VFF constructs, information about SPION fate in the engineered implant is of key importance. In our study, SPIONs remained within VFF for 40 days, whereby considering normal cell division, the cells were split in culture every five days. Therefore, we estimate that the iron will vanish gradually from the constructs, either by cellular iron metabolism or via a dilution process by cell division. Independent of the clearance mechanism, it is important to determine whether and how long the pre-loaded SPIONs will remain within the cell.

### Magnetic Cell Guidance in 2D

**Fig. 5:**
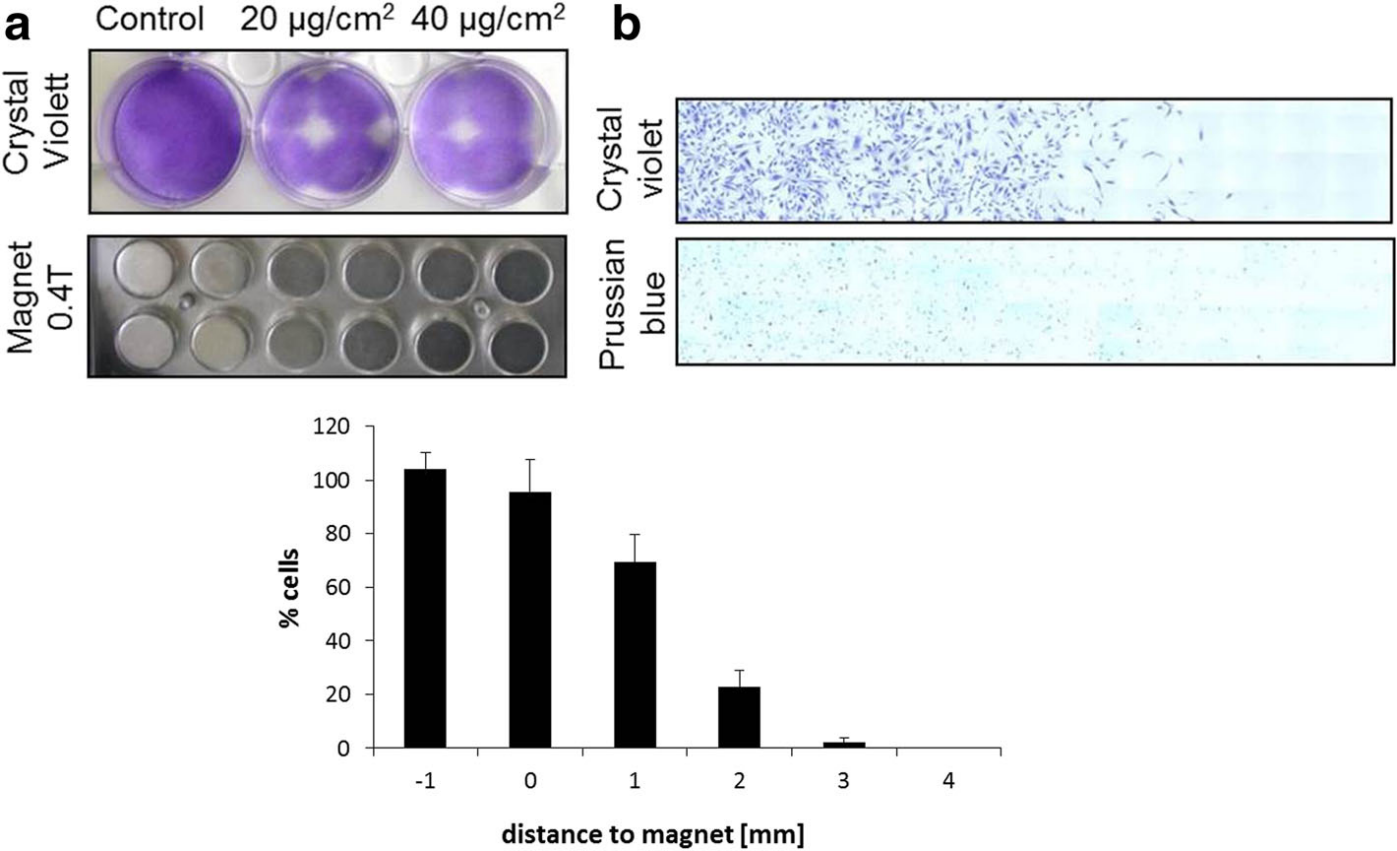
2D magnetic guidance of VFF. **a** Magnetic guidance of SPION-loaded VFF shows cells only growing in areas where the magnet is present. *Upper*: VFF loaded with 0 (control), 20, or 40 μg/cm^2^ SPIONs seeded in a 6-well plate, which was placed on top of a 24-well magnet plate (*lower*). (**b**) Crystal violet (*upper*) and Prussian blue (*lower*) staining of VFF grown over the magnet (*left*) and in magnet-free zone (*right*). Quantitative analysis: % of cells measured in 4 mm distance of the magnet

To ascertain that magnetized cells can actually be directed in a magnetic field, VFF loaded with 20 and 40 μg/cm^2^ of SPIONs were seeded in a 6-well plate placed on top of a 24-well magnet plate (Fig. 5a). While control cells grow uniformly distributed within the 6-well plate, cells loaded with SPIONs accumulate particularly in the regions where the magnet was present. In contrast, the well areas without a magnet below were cell-free. Cells were stained with crystal violet to ascertain that cell-abundant areas are clearly located above the magnet and Prussian blue staining verified iron uptake of VFFs in this experiment (Fig. 5b). Quantitative analysis revealed that within 2-mm distance from the magnet, cell attachment decreases to fewer than 30% (22 ± 6%) and within 3 mm fewer than 5% (2 ± 2%). With this proof of concept, we demonstrated the feasibility of MTE using SPIONs for VFF cell guidance in 2D.

As MTE has already been effectively applied for the development of 3D co-culture systems, which will be indispensable for the future bioengineered VFs [35], our next goal is to establish co-culture systems, in order to generate a functional VF implant. In this study, we used VFFs; assuming that being the major extracellular matrix (ECM) producers, these cells are ideally suited for 3D tissue formation of the VF. Since this ECM is very special in vocal folds, we anticipate that the co-culture with epithelial cells may also be necessary to stimulate VFFs towards production of the ECM needed for normal VF function. Future experiments will thus address the evaluation of effective SPION loading to create 3D-constructs in specific shapes depending on the magnet used as well as the establishment of co-culture systems with epithelial cells, whereupon functionality will be proven in a flow channel model of the rabbit larynx.

Taken together, knowledge provided by this study will particularly support the next experiments where prove of concept of magnetization in the formation of 3D constructs under magnetic guidance must be established. The goal lies within a compromise between sufficient SPION loading for an adequate magnetic controllability and keeping cellular functions intact, without interfering with normal cell behavior especially concerning vocal cord vibration. These investigations are the basis for future magnetic tissue engineering of the vocal fold.

## Conclusions

Nanotechnology is of high potential for various medical applications, including cancer therapy [36], imaging [37], or tissue engineering [38]. However, just as any new technology, the use of nanoparticles in humans has concomitant risks. Hence, nanotoxicologic considerations must be given proper attention before introducing new methods in the clinic [39]. SPIONs used in this study were synthesized and characterized extensively before [40–42] and have already been proven effective for drug delivery in cancer therapy [14, 43]. In our previous studies, we have evaluated the effects of SPIONs on VFF viability and their cellular uptake, revealing no evidence of toxicity in concentrations used [30, 41, 44]. Since the aim of developing a 3D bioengineered vocal fold is very ambitious, several milestones have to be accomplished during the course of the studies. Consequently, our next step was to investigate the influence of SPIONs on cell behavior relevant for tissue engineering as well as the iron clearance from pre-loaded VFFs.

Cell features including adhesion, spreading, and migration, which are essential to function normally during 3D cell formation of VFF transplants, were proven to be intact in a dose-dependent manner after SPION treatment, revealing a convenient usage of MTE to develop 3D VFF constructs. After 40 days, cells were free of iron either by metabolization or by rarefication during cell division; therefore, we conclude no influence of iron nanoparticles on vibratory functions of the bioengineered vocal cord when using 20 μg/cm^2^. Furthermore, data on 2D cell culture revealed that magnetisized VFFs can be controlled to grow only where a magnetic field is present as a first proof of concept.

Our results will constitute a solid basis for a successful transfer of this technique into humans, in order to provide an individual and personalized VF implant.

## Abbreviations

3D: Three dimensional
DMEM: Dulbecco’s modified Eagle’s medium
ECM: Extracellular matrix
FCS: Fetal calf serum
H_2_O ddist: Double-distilled water
MP-AES: Microwave plasma-atom emission spectroscopy
MTE: Magnetic tissue engineering
PBS: Phosphate-buffered saline
PI3K: Phosphoinositid-3-kinase
SPION: Superparamagnetic iron oxide nanoparticles
VF: Vocal fold
VFF: Vocal fold fibroblasts
